# Effects of Nisin, Cecropin, and *Penthorum chinense* Pursh on the Intestinal Microbiome of Common Carp (*Cyprinus carpio*)

**DOI:** 10.3389/fnut.2021.729437

**Published:** 2021-10-21

**Authors:** Famin Ke, Peijuan Xie, Yanrong Yang, Liu Yan, Ailing Guo, Jian Yang, Jing Zhang, Li Liu, Qin Wang, Xiaowei Gao

**Affiliations:** ^1^School of Pharmacy, Southwest Medical University, Luzhou, China; ^2^Department of Pharmacy, The Affiliated Hospital of Southwest Medical University, Luzhou, China; ^3^Department of Chemistry, Zhejiang University, Hangzhou, China

**Keywords:** nisin, cecropin, *Penthorum chinense* pursh, common carp, intestinal microbiome, feed additives

## Abstract

Following a ban on antibiotic use in the feed industry, trials on the effects of various immunostimulants (prebiotics, probiotics, antimicrobial peptides [AMPs], and herbs) on the survival, growth, immunity, and disease control of farmed fish in aquaculture are being rapidly conducted. The wide variety of microbes with roles in nutrition, metabolism, and immunity in the fish intestine is the primary factor affecting the fermentability and functionality of dietary immunostimulants. For this reason, the dynamic interactions between immunostimulants and the intestinal microbiome may influence fish health. In this study, the effects of two agriculturally important AMPs (nisin and cecropin) and one herb (*Penthorum chinense*) on the gut microbiome of common carp were investigated, using 16S rDNA high-throughput sequencing. The results suggest that all three substances can alter the richness, diversity, and composition of the intestinal microbiota of common carp. *P. chinense* had a similar effect on the gut microbiota of common carp to that of nisin, and both promoted more striking changes in the gut microbiota community than did cecropin. The relative abundance of Proteobacteria was lower in the nisin and *P. chinense* groups than in the control and cecropin groups. The relative abundance of Bacteroidetes in the nisin, cecropin, and *P. chinense* groups was markedly increased, compared with that of the control group. Additionally, nisin, cecropin, and *P. chinense* showed obvious anti-inflammatory effects on the fish intestine, which was reflected by significantly increasing the expression levels of two anti-inflammatory cytokines IL-10 and TGF-β. Some digestive enzyme activities in the fish intestine were also significantly enhanced by supplementing these three substances in feeds.

## Introduction

Fishes are one of the main sources of consumable proteins for humans. Aquaculture has been growing rapidly in recent years and has now overtaken capture fisheries as the main way for humans to obtain food fish. With the increased use of intensive and high stocking-density breeding in fish farming, the risk of disease outbreaks has increased significantly (1). To avoid economic losses caused by bacterial infections, antibiotics and chemotherapeutics have been widely used as feed additives to prevent and treat diseases in fish (2). However, studies have demonstrated that supplementing antibiotics in feeds may have negative effects on fish health, including oxidative stress, immunosuppression, and histopathological damage (3–5). In addition, overuse of antibiotics results in many side effects that threaten human health, including presence of undesired drug residues in the food chain, development, and enrichment of drug-resistant genes, and emergence of drug-resistant bacteria (6). Studies have projected that antibiotic resistance will cause 10 million deaths and $100 trillion loss in the US by 2050 if this problem remains unsolved (7). Owing to their negative effects, antibiotics used as feed additives and growth promoters in the feed industry have been banned by the European Union (EU) since 1997 (8).

To achieve sustainable development in aquaculture and provide fish products for safe and nutritious human consumption, developing alternatives to antibiotic feed additives is urgent and economically significant. Antimicrobial peptides (AMPs) are small peptide fragments with broad-spectrum antimicrobial activity, including antibacterial, antifungal, antiviral, and anti-carcinogenic properties (9). AMPs are a key component of the innate immune system of almost all organisms, and more than 2,600 AMPs have been identified to date (10). Owing to their lack of drug resistance potential, AMPs have been considered excellent alternatives to antibiotics in both the medical and feed industries. When used as feed additives, some AMPs have been shown to expedite the growth of fishes and increase the resistance of fishes to pathogens (11). However, several studies suggest that different types or concentrations of AMPs have different effects on fish health and performance when used as feed additives for specific fish species (12). Thus, expanding our knowledge regarding the effects of AMPs on different fish will help us to develop suitable AMP-based feed additives for particular fish species. Additionally, some immunostimulants, including probiotics, prebiotics, and herbs, have also shown positive effects on fish performance, disease prevention, and disease control when supplemented as feed additives (2). Several studies have investigated the effects of these reagents on growth, antioxidant and immune responses, and disease resistance in fishes, but effects on the intestinal microbiome have rarely been examined. The intestinal microbiome—a large, diverse, complex, and dynamic community of microbes that colonize the fish gut—is receiving increased attention owing to its important roles in host nutrient digestion, energy metabolism and absorption, immunity, pathogen resistance, and health maintenance (13). The composition of the fish gut microbiota is influenced by many endogenous and exogenous factors, including genetics, phylogeny, sex, body weight, age, immunity, diet, and habitat. Maintaining a normal healthy gut microbiota is essential for fish health.

Nisin is a ribosome-synthesized AMP that is naturally produced by the probiotic bacterium *Lactococcus lactis*. Post-translational modifications are needed to generate mature nisin, which consists of 34 amino acid residues containing dehydrobutyrine, dehydroalanine, one lanthionine, and four methyl-lanthionine rings (9). Nisin shows good inhibitory activity against gram-positive bacteria, including the foodborne pathogen *Listeria monocytogenes*. Owing to its “generally regarded as safe” (GRAS) status, nisin has been widely approved in the food industry by many official organizations, including the World Health Organization (WHO), the European Food Safety Authority (EFSA), and the United States Food and Drug Administration (FDA) (9). It has been reported that dietary nisin can modulate the gut microbial community and enhance the growth performance of broiler chickens (14). Cecropin, naturally generated by the moth *Hyalophora cecropia*, is a strongly cationic, amphipathic AMP with broad-spectrum antimicrobial activities (15). When used as feed additives, members of the cecropin family have shown the capacity to increase animal performance and resistance to lethal pathogen infection. Dietary cecropin increases nitrogen retention and crude fat digestibility; it also enhances apparent metabolizable energy content in broilers (16). Feeding cecropin improved the villus-height-to-crypt-depth ratio in the jejunum and ileum in piglets challenged with *Escherichia coli*, compared with that of controls (17).

Many Chinese medicinal herbs have been investigated as alternatives to antibiotics in the feed industry. When used in fish aquaculture, herbs have shown immunomodulatory, antiviral, antibacterial, and antiparasitic activities and can stimulate appetite and promote weight gain (18, 19). *Penthorum chinense* Pursh is a traditional Chinese medicine that alleviates heat and diuresis, causes detoxification, and promotes blood circulation (20). It is now widely used to treat several liver diseases, such as non-alcoholic and alcoholic fatty liver disease, as well as infectious hepatitis. The extract of *P. chinense* has strong antioxidant and anticomplement activities and can inhibit the growth of many pathogens, including *Staphylococcus aureus, Staphylococcus epidermidis*, and *Pseudomonas aeruginosa* (21). Additionally, a recent study demonstrated that *P. chinense* can alter the structure of the gut microbial community in mice and increase the abundance of some probiotics (22). At present, the application of *P. chinense* in aquaculture has not been documented. We assumed that the properties of *P. chinense* mentioned above may have positive effects when used as feed additives in aquaculture.

The common carp (*Cyprinus carpio* L., family *Cyprinidae*) is an omnivorous and stomachless fish. It represents the fourth most-produced fish species in aquaculture worldwide and is produced mainly in Asia and Europe (23). The effects of AMPs and certain herbs as feed additives on common carp have been reported previously; however, these studies mainly assessed and compared the changes in fish performance, blood biochemical parameters, antioxidant activity, and immunity between experimental and control groups (2, 24–30). Considering that feed additives in the fish diet are administered orally, they inevitably enter the gut and directly interact with fish intestinal microflora. Understanding the composition of the fish intestinal microbiota and the changes caused by feed additives in diets will guide the development of novel additives to improve the health and performance of fishes. Here, we report the effects of nisin, cecropin, and *P. chinense* on the gut microbiome of common carp, and we evaluate their effects on gut health and digestive enzyme activities in this species.

## Materials and Methods

### Experimental Fish and Diet Preparation

Common carp used in this study were obtained from a fish farm in Luzhou City, Sichuan Province, China. Initially, 120 healthy sub-adult fish of similar size (296.75 ± 7.22 g) were randomly distributed into four groups. Each group was housed in six plastic 200 L tanks (80 × 50 × 50 cm) with five individuals per tank. Before the feeding trial, fish were acclimatized for 2 weeks with the control diet. Commercially available nisin (amino acid sequence: ITSISLCTPGCKTGALMGCNMKTATCHCSIHVSK) was purchased from Hengkang Food Additive Co., Ltd. (Tianjin, China). Cecropin (amino acid sequence: KWKLFKKIEKVGQNIRDGIIKAGPAVAVVGQATQIAK) was obtained from Shandong Ruitai Biotechnology Co., Ltd. (Shandong, China). The stem of *P. chinense* was purchased from Sichuan Jiyuntang Traditional Chinese Medicine Co., Ltd., and crushed into powder using a high-speed universal grinder after freezing in liquid nitrogen. A commercial basal diet for common carp was used as the control diet (CON), and experimental diets were prepared by mixing nisin (0.05%, NIS), cecropin (0.01%, CEC), or *P. chinense* (4%, PCH) powder into the control diet, according to the manufacturers' instructions. The control diet used in this experiment contained 16.0% fish meal (FM), 34.0% soybean meal, 4.0% corn oil, 9.0% wheat bran, 11.0% wheat flour, 7.0% dextrin, 14.0% corn gluten, 3.0% Ca(H_2_PO_4_)_2_, 0.1% vitamin mixture, and 1.0% mineral mixture. For the feed preparation, basal diets were completely ground, mixed with the corresponding additive powder, made into sinking pellets at room temperature, and stored at −20°C until use.

### Experimental Design and Feeding

Experiments in this study were reviewed and approved by the Southwest Medical University Ethics Committee and performed at Southwest Medical University (Luzhou, China). During the experiment, the four groups of fish were fed twice daily with their corresponding diets at 08:00 a.m. and 18:00 p.m. Half of the water in the tanks was changed daily with tap water to maintain water quality. The water parameters were recorded and were as follows: temperature ranged from 20 to 28°C; dissolved oxygen concentration ranged from 5.2 to 6.1 mg/L; pH ranged from 7.1 to 7.9. The feeding experiment lasted for four consecutive weeks under a 12 h light/12 h dark photoperiod. The fish in each group were weighed at the beginning of the feeding experiment and at the end. Weight gain was calculated by subtracting the starting weight from the final weight.

### Isolation of Intestinal Bacterial DNA and Sequencing

After the feeding experiment, we randomly selected one fish per tank, and a total of six fish from each group were used for gut microbiome analysis. After euthanasia with tricaine methanesulfonate (MS-222, Sigma, USA), the sampled fish were washed with 70% ethanol and sterile water and then dissected, and the intestinal tissues were aseptically removed from the abdominal cavity. Then, the gut contents were gently squeezed out and harvested separately into sterile tubes. After freezing in liquid nitrogen, the gut content samples were stored at −80°C until use.

Bacterial DNA was extracted from the fish gut content samples directly using the E.Z.N.A. Stool DNA kit (OMEGA, Bio-tek, USA). The purity and concentration of the resulting DNA in each sample were determined using 1% agarose gel electrophoresis and a NanoDrop^TM^ instrument (Thermo Fisher Scientific, Wilmington, DE, USA), respectively. The V4/V5 hypervariable regions of the bacterial 16S rRNA gene were amplified using polymerase chain reaction (PCR) with the extracted genomic DNA as a template. The primer pairs used in the PCR were 338f and 806r (Table 1). The PCR mixture and reaction conditions were set as previously described (13). In brief, the PCR mixture consisted of 9.0 μl ddH_2_O, 2.5 μl PCR buffer (10×), 1.0 μl KOD-Plus-Neo DNA polymerase, 2.5 μl dNTPs, 1 μl 338f/806r primers, and 2 μl genomic DNA template. PCR conditions were set as: 95°C for 5 min; 30 cycles of 95°C for 30 s, 55°C for 30 s, and 68°C for 30 s; and a final extension at 68°C for 5 min. After electrophoresis on a 1% agarose gel, the PCR products were purified, and the sequencing libraries were constructed and sequenced as described previously (13).

**Table 1:** Primers sequences, amplification temperature, and amplicon information for qRT-PCR.

**Primer**	**Sequence (5^′^-3^′^)**	**Annealing temperature (^°^C)**	**Amplicon size (bp)**	**Accession no**.
338-f	ACTCCTACGGGAGGCAGCAG			
806-r	GGACTACHVGGGTWTCTAAT			
IL1β-f	AAGGAGGCCAGTGGCTCTGT	58	69	AB010701
IL1β-r	CCTGAAGAAGAGGAGGCTGTCA			
TNFα-f	GCTGTCTGCTTCACGCTCAA	58	106	AJ311800
TNFα-r	CCTTGGAAGTGACATTTGCTTTT			
IL10-f	GCTGTCACGTCATGAACGAGAT	58	132	AB110780
IL10-r	CCCGCTTGAGATCCTGAAATAT			
TGFβ-f	ACGCTTTATTCCCAACCAAA	58	95	AF136947
TGFβ-r	GAAATCCTTGCTCTGCCTCA			
β-actin-f	GAAGTGTGGTGTGGACATCCGTAA	58	247	JQ619774.1
β-actin-r	AGACTCATCGTACTCCTGCTTGCT			

### Sequence Processing and Bioinformatic Analysis

The sequencing raw reads obtained in this study were deposited into the National Centre for Biotechnology Information short read archive database under the accession number PRJNA738662. After sequencing, the resulting sequences were processed and quality-filtered to remove low-quality reads using the TrimGalore software and merged using FLASH 2, as previously described (13). Sequences with a quality score <20, ambiguous bases, mismatches in barcodes, or primers with more than 2 bases were removed. To clarify the bacterial community compositions of each group, operational taxonomic unit (OTU) analysis was performed using the Quantitative Insights Into Microbial Ecology (QIIME) toolkit (version 1.17). According to currently accepted standards for prokaryotic species, OTUs were clustered with a threshold of 97% similarity, and chimeric sequences were identified and removed as described previously (9). With a confidence threshold of 70%, the representative OTUs were annotated with taxonomic information using the SILVA ribosomal RNA gene database.

Bioinformatic analysis was performed using the i-Sanger Cloud Platform (www.i-sanger.com). Rarefaction curves were generated by plotting the number of OTUs against the number of identified sequences. The Sobs, Chao, Good's coverage, Ace, Shannon, and Simpson indices that reflected the α-diversity of each group were determined using the Mothur software. The Sobs, ACE, and Chao indices were used to reflect the richness of bacterial species in a community regardless of the abundance of each species, whereas the Shannon and Simpson indices were used to reflect species richness and species evenness. β-diversity analysis, which reflects community diversity and species differences among the four groups, was assessed by weighted UniFrac-based principal coordinate analysis (PCoA) and hierarchical clustering trees using the R software package. The Wilcoxon rank-sum test were used to determine statistically significant differences in microbial composition at the genus level between the control and the other three experimental groups. The main differences in the species level among the four groups were revealed using hierarchical clustering heatmap analysis.

### Quantitative Reverse Transcription-PCR (qRT-PCR) Analysis

qRT-PCR was used to assess the transcriptional levels of target genes. Total RNA was extracted from the intestinal tissues of the sampled fish using the TRIzol method with an RNA Isolation Plus kit (Takara, Dalian, China) according to the manufacturer's instructions. After verification of the integrity and purity of the total RNA using 1.5% agarose gel electrophoresis, cDNA was obtained by reverse-transcribing RNA with a HiScript® Reverse Transcriptase Kit (Vazyme, Jiangsu, China). The primers used in this study are listed in Table 1. qRT-PCR was performed using a Real-Time PCR Detection System (Bio-Rad, USA) with the AceQ™ qPCR SYBR® Green Master Mix kit. The PCR program was set up as follows: 95°C for 10 min, followed by 45 cycles of 95°C for 15 s, 58°C for 30 s, and 72°C for 30 s. The relative mRNA levels of target genes were normalized using β-actin as an internal standard with the 2^−Δ*ΔCt*^ method (31).

### Enzyme Activity Analysis

Six fish in each group were randomly selected for digestive enzyme activity analysis. The digestive enzyme activities in the intestines of the sampled fish were determined using commercial assay kits (Jiancheng Bioengineering Company, Nanjing, China). After freezing in liquid nitrogen, intestinal samples were ground into a powder and homogenized in phosphate buffer (pH 7.4) on ice. Thereafter, the resulting samples were centrifuged at 4,000 × *g* for 10 min, and the supernatant fraction was collected. After quantification of the soluble protein concentrations using the Bradford method, the activities of amylase, lipase, and protease in each sample were analyzed. One unit of amylase activity was defined as the amount of the enzyme required to hydrolyze 10 mg starch in 30 min. One unit of lipase activity was defined as the amount of the enzyme required to hydrolyze triglyceride substrate to form 1 μmol glycerol. One unit of protease activity was defined as the amount of the enzyme required to release 1 mg tyrosine from the substrate per min.

### Statistical Analysis

Unless otherwise indicated, statistical analysis was performed in GraphPad Prism 8.0 software (USA) by one-way ANOVA with Dunnett's test (for comparison between two groups) or *post hoc* Tukey-kramer test (for comparison among three or more groups). All data were considered statistically significant at *p* < 0.05.

## Results

### Growth Performance

**Figure 1:**
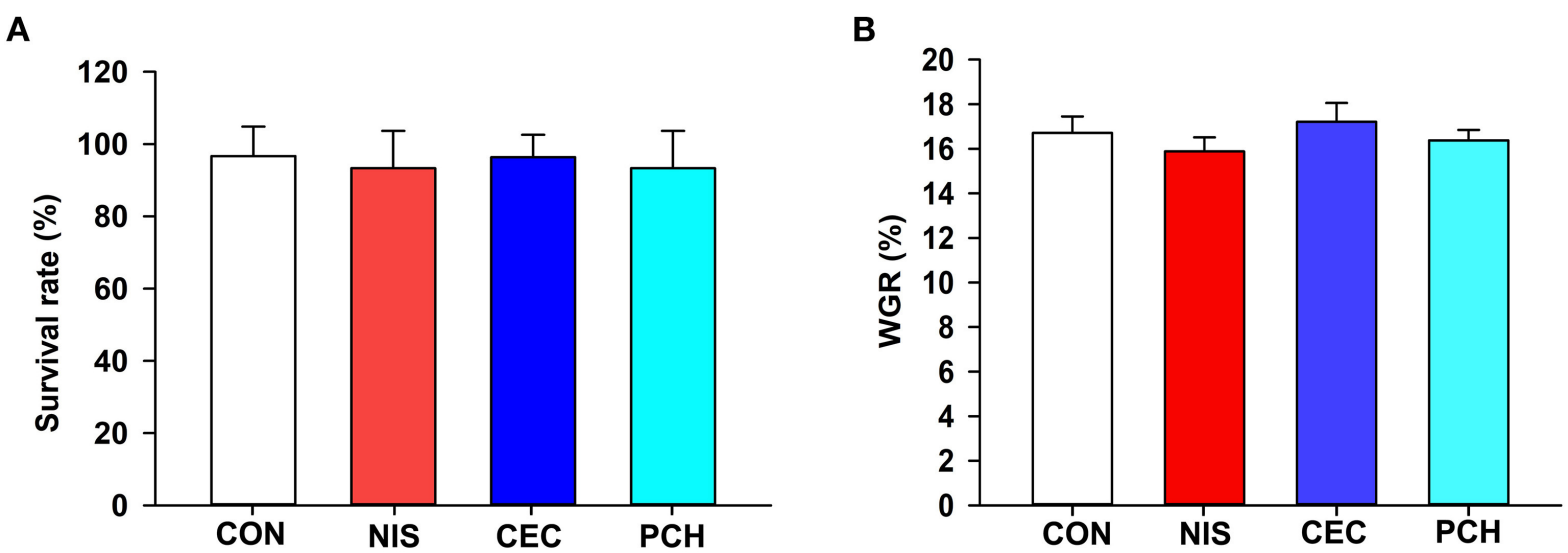
Effects of the three additives on the growth performance of common carp. **(A)** Survival rate of the common carp in the four groups. **(B)** Weight gain rate (WGR) of the common carp in the four groups. WGR is calculated as: WGR= (final weight–initial weight) × 100/(initial weight). No significant differences were observed among the four groups.

The results of growth performance of common carp at the end of the experimental trials are shown in Figure 1. There were no significant differences in survival rate or body weight gain values among the four groups, indicating that the three additives used in this study displayed negligible effects on the growth performance of common carp.

### Characteristics of the High-Throughput Sequencing Data

**Figure 2:**
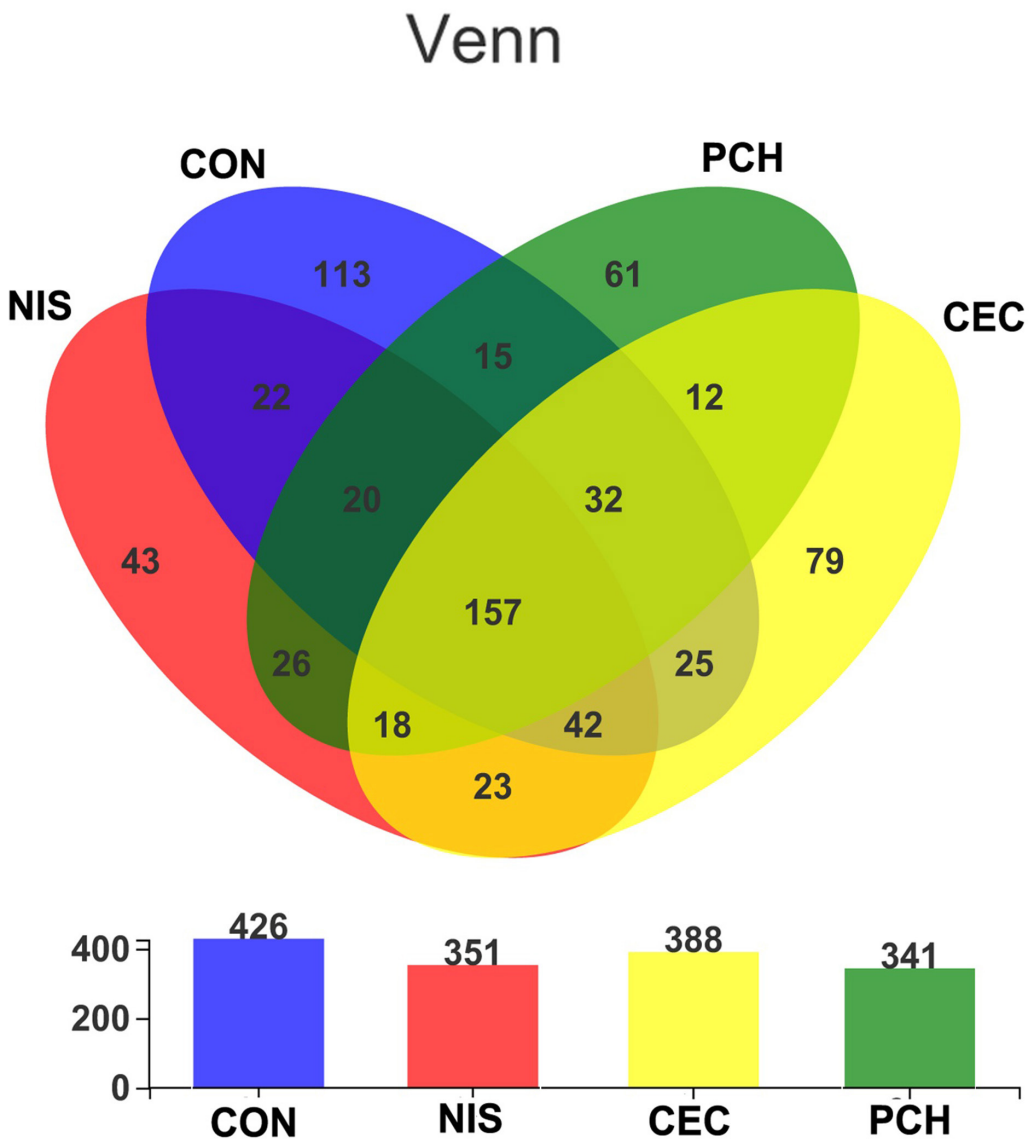
The number of shared and unique operational taxonomic units (OTUs) in different samples. Venn diagrams showing the numbers of unique and shared OTUs among the four groups. Overlaps in the upper panel represent the shared OTUs of corresponding sample groups. The total number of OTUs in each group (*n* = *6*) is shown in the lower panel.

A total of 1,182,758 valid sequences were obtained after removing low-quality reads according to the criteria mentioned in subsection Sequence processing and bioinformatic analysis of “Materials and methods.” In each sample, the sequence Good's coverage index was ≥99%, and the rarefaction curve reached a saturation plateau (Supplementary Table 1 and Supplementary Figure 1). These results suggest that the sequencing depth was adequate, and most bacterial phylotypes could be identified. With a 97% sequence similarity cut-off threshold, the obtained valid sequences were delineated into 688 OTUs. The total OTU numbers of the CON, NIS, CEC, and PCH groups were 426, 351, 341, and 388, respectively. The number of shared OTUs in the four groups was 157, whereas the number of unique OTUs was 113 in the CON group, 43 in the NIS group, 79 in the CEC group, and 61 in the PCH group (Figure 2).

### Diversity Analysis of the Microbial Community

**Figure 3:**
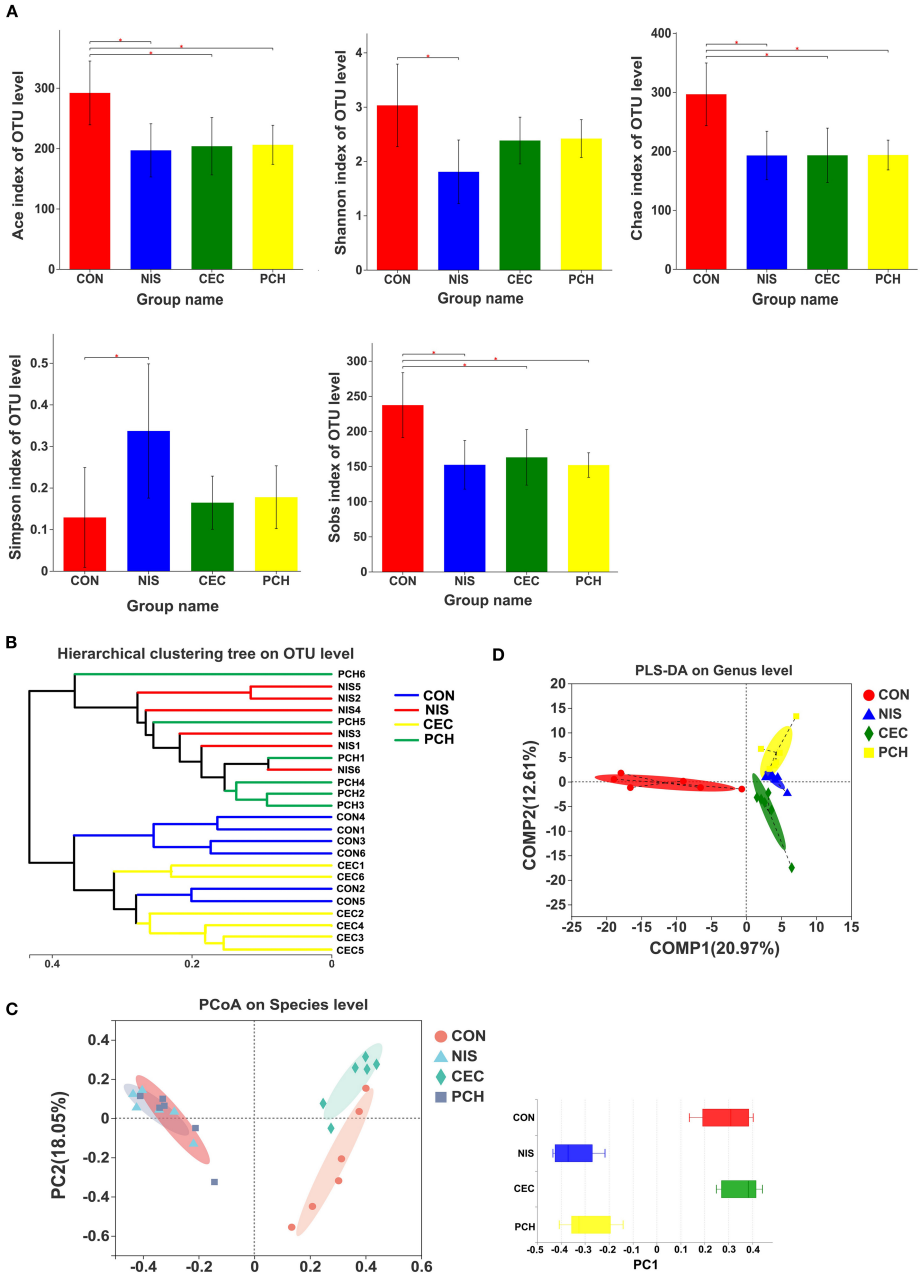
Diversity analysis of the gut microbial communities among the four groups. **(A)** The α-diversity of gut microbial communities is significantly different between the control and the three experimental groups. Bacterial community diversity and richness were presented as the Shannon, Simpson, ACE, Chao, and Sobs indices. One-way ANOVA with Dunnett's test was used for statistical analysis to determine significant differences between the control and the three experimental groups. *p* < 0.05 was considered statistically significant (*p < 0.05). **(B)** Hierarchical clustering tree of Bray-Curtis distances. Each branch represents one fish gut bacterial community. **(C)** Principal coordinate analysis (PCoA) of the four groups. Principal component 1 (PC1) and 2 (PC2) explained 40.7 and 18.05% of the variance, respectively. Distances between each symbol in the plot reflect relative dissimilarities of relative bacterial communities. Discrete degree of PC1 is shown in the right panel. **(D)** Partial least squares discriminant analysis (PLS-DA) of the four groups.

The α- and β-diversity of the microbial communities of the four groups were analyzed separately to determine the effects of nisin, cecropin, and *P. chinense*, when used as feed additives, on the diversity of intestinal microbial communities of common carp. The richness and community diversity indices of each group were calculated and compared. As shown in Figure 3A, the Sobs, ACE, and Chao indices were significantly lower in the three experimental groups than in the control group, indicating that nisin, cecropin, and *P. chinense* as feed additives reduced the species richness of the intestinal microbiota in common carp. The Shannon index was higher in the control group than in the three experimental groups, whereas the Simpson index showed the opposite trend.

According to the hierarchical clustering tree shown in Figure 3B, the cecropin group was clustered together with the control group, whereas the nisin group and the *P. chinense* group, clustered together, were located on different branches of the tree. PCoA analysis was performed to compare the bacterial communities among the four groups (Figure 3C). The weighted UniFrac distance was used to determine the PCoA score plot, and each symbol in the plot represents one sample. The discrete degree of PC1 is also shown. The two principal coordinates accounted for 58.75% of the total variation between the groups. The nisin and *P. chinense* groups were clustered together to the right of the plot and located far away from the control group, whereas the cecropin group was located near, and showed only slight dissimilarity with the control group. A structural rearrangement of the gut microbiota of common carp in the three experimental groups was also detected by partial least squares discriminant analysis, as shown in Figure 3D.

### Composition Analysis of the Microbial Community

**Figure 4:**
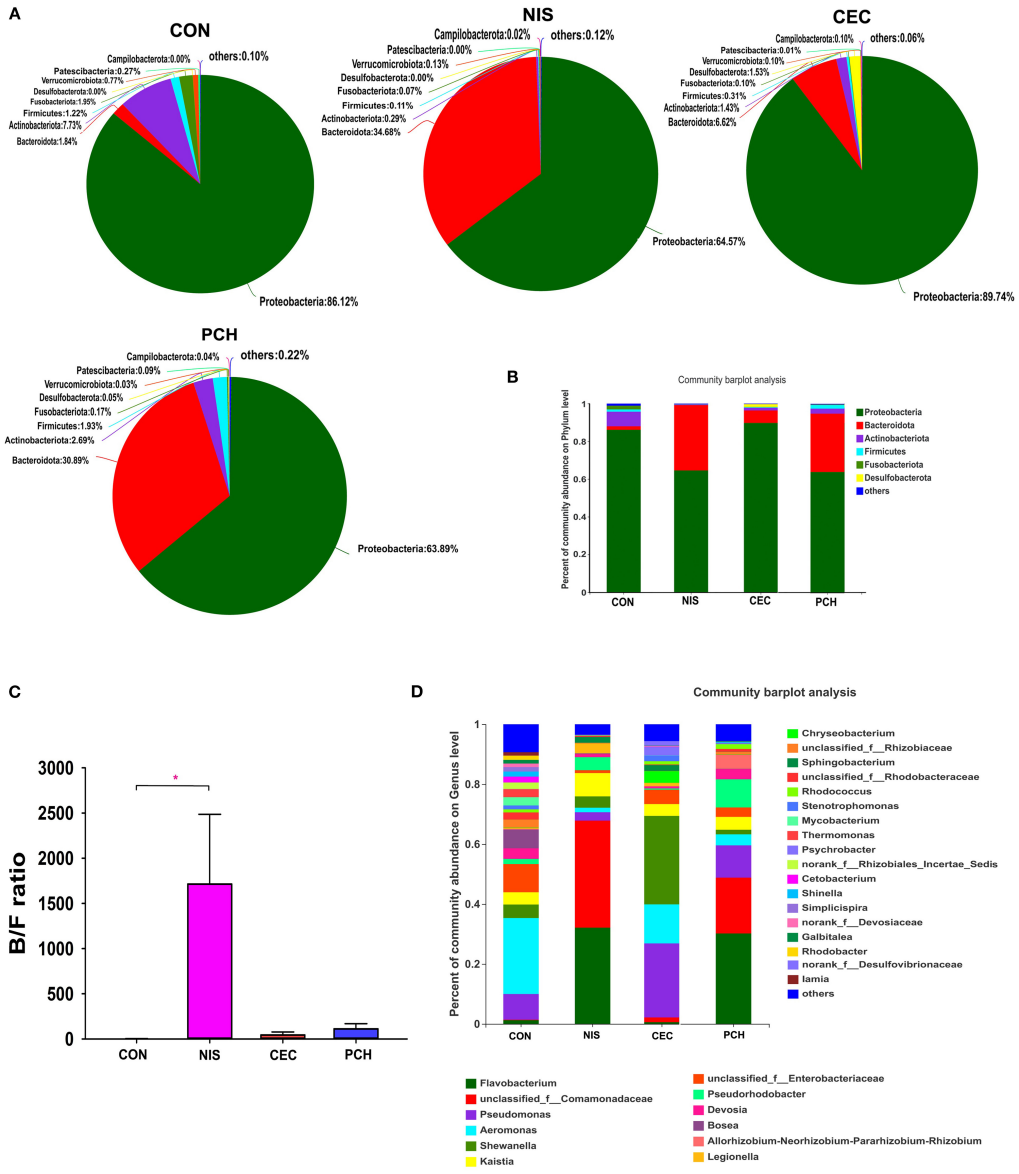
Distribution of bacterial phyla and genera among the four groups. **(A)** Pie diagrams show the bacterial composition of the four groups at the phylum level. The relative abundance of bacterial phyla in each group is shown. **(B)** Column diagrams of distribution of bacterial phyla among the four groups. **(C)** Ratio of Bacteroidetes/Firmicutes (B/F) in the four groups. Data are expressed as mean with SD (*n* = *6*). *p* < 0.05 was considered statistically significant (*p < 0.05). **(D)** Column diagrams of distribution of bacterial genera among the four groups. “Others” refers to bacteria with < 0.01% abundance.

A total of 688 OTUs obtained in this study could be assigned to 23 bacterial phyla based on phylogenetic information. The distribution of bacterial phyla among the four groups is presented by both pie and column diagrams in Figures 4A,B, and the relative abundance of the bacterial phyla in each group is also shown. Proteobacteria was the most abundant phylum in all four groups, and the relative abundance of this phylum in the CON, CEC, NIS, and PCH groups was 86.12, 89.74, 64.57, and 63.89%, respectively, indicating that the relative abundance of Proteobacteria was lower in the NIS and PCH groups than in the control group. The relative abundance of Bacteroidetes in the NIS, PCH, and CEC groups was significantly increased: 1.84% (CON), 6.62% (CEC), 34.68% (NIS), and 30.89% (PCH), when compared with that of the control group, leading to an increase in the Bacteroidota/Firmicutes (B/F) ratio in the three experimental groups (Figure 4C). The phylum Desulfobacterota, which was absent in the CON group, was present in the CEC and PCH groups.

The number of bacterial genera identified from the four groups was 399, and the relative abundance of each bacterial genus is shown in Figure 4D. The five most abundant genera in the control group were *Aeromonas* (25.35%), *Enterobacteriaceae* (9.38%), *Pseudomonas* (8.56%), *Bosea* (6.25%), and *Shewanella* (4.49%), whereas the top five genera in the nisin group were *Comamonadaceae* (35.73%), *Flavobacterium* (32.11%), *Kaistia* (7.79%), *Pseudorhodobacter* (4.37%), and *Shewanella* (3.79%) (Supplementary Figure 2). The five most abundant genera in the cecropin group were *Shewanella* (29.53%), *Pseudomonas* (24.69%), *Aeromonas* (13.03%), *Enterobacteriaceae* (4.79%), and *Bosea* (3.94%), and the top five genera in the *P. chinense* group were *Flavobacterium* (30.2%), *Comamonadaceae* (18.62%), *Pseudomonas* (10.79%), *Pseudorhodobacter* (9.4%), and *Rhizobium* (4.70%). The relative abundance of each bacterial genus in the individual fish is shown in Supplementary Figure 3.

### Analysis of Overall Changes in Microbial Community

**Figure 5:**
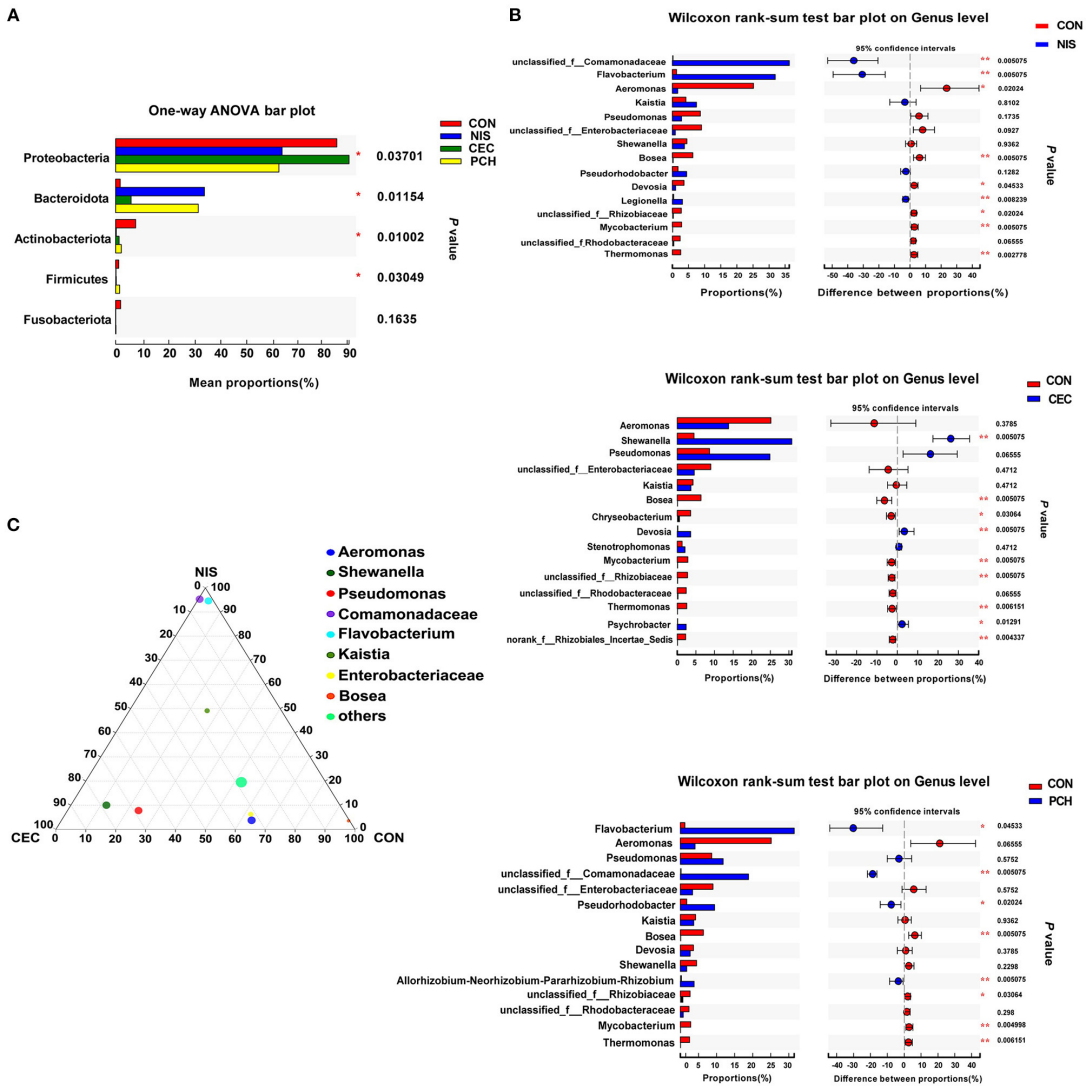
Analysis of the differences in specific microbial taxa among the four groups. **(A)** The differences of microbial composition across the four groups at the phylum level. One-way ANOVA with a *post hoc* Tukey-kramer test was used for statistical analysis to determine significant difference among the four groups; **(B)** Wilcoxon rank-sum analysis of the differences between the control (CON) and the three experimental groups at the genus level. Data are presented as mean ± SD (*n* = 6). *p* < 0.05 was considered statistically significant (*p < 0.05, **p < 0.01). **(C)** Ternary plot analysis of differences in the main genera in CON, nisin (NIS), and cecropin (CEC) groups. Dot size indicates the relative abundance of the genera.

Significant differences in the gut microbiome composition among the four groups at the phylum level were shown in Figure 5A. Significant variations in the relative abundance of Proteobacteria (*p* = 0.03701), Bacteroidetes (*p* = 0.01154), Actinobacteria (*p* = 0.01002), and Firmicutes (*p* = 0.03049) were observed. The CEC, NIS, and PCH groups increased in the relative abundance of Bacteroides and decreased in the relative abundance of Actinobacteriota and Fusobacteriota, compared with those in the control group. The relative abundance of Proteobacteria significantly decreased in the NIS and PCH groups, and the relative abundance of Firmicutes significantly decreased in the NIS and CEC groups.

**Figure 6:**
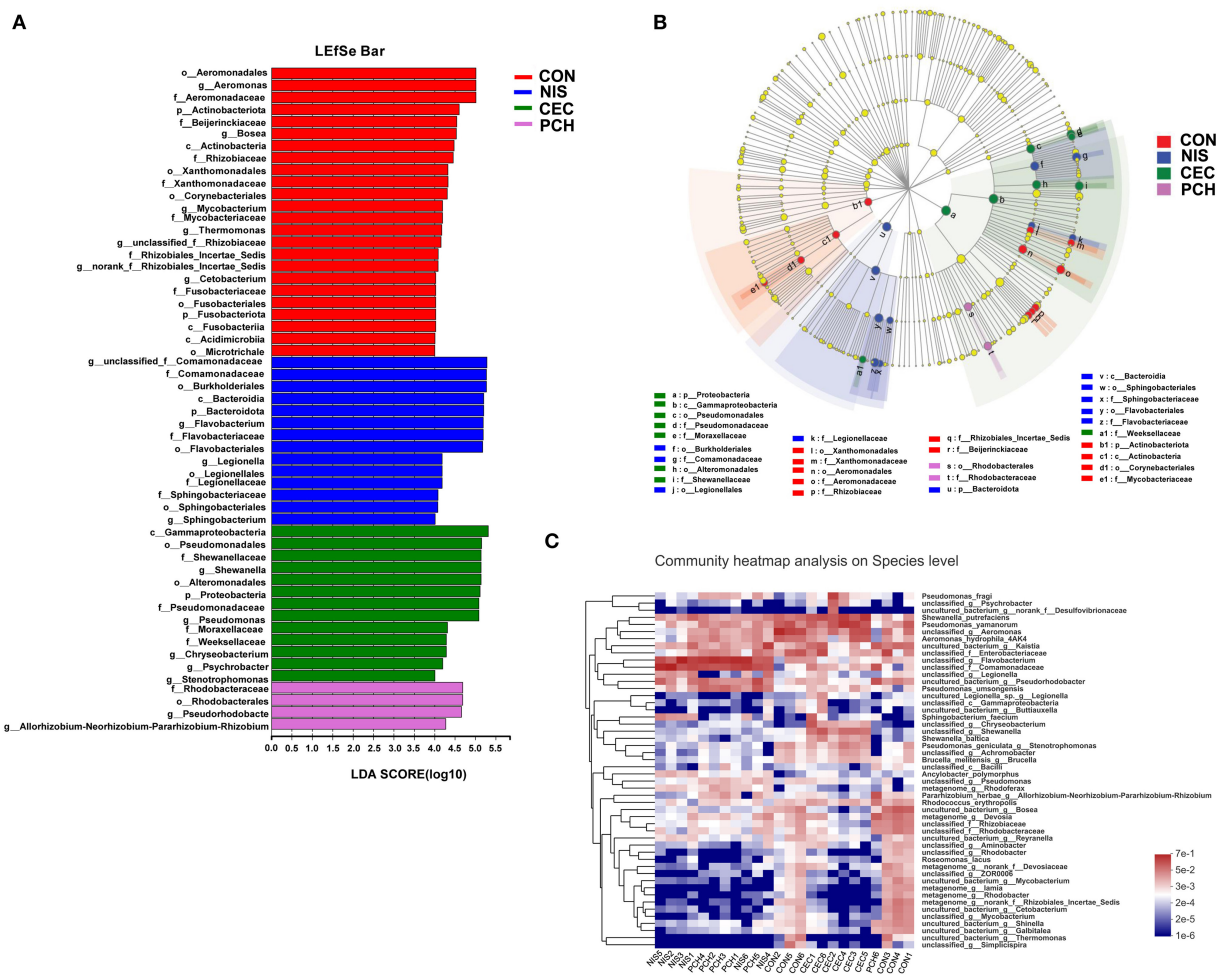
LEfSe analysis of intestinal microbiota communities of the four groups. **(A)** Linear discriminant analysis (LDA) score in the four groups. Histogram showing differentially abundant taxa (LDA score > 4) in the gut microbiota of the four groups. The length of the column is proportional to the taxa abundance. **(B)** LDA effect size (LEfSe) analysis in the four groups. p, phylum; c, class; o, order; f, family; g, genus. **(C)** Heatmap of gut bacteria species from the four groups. The bacterial phylogenetic tree (shown on the left of the figure) was generated using the neighbor-joining method. The relative abundance values of bacterial species in different samples are depicted by color intensity, and the color legend corresponding to different values is to the right of the figure.

Using the Wilcoxon rank-sum test, significant variations in the gut microbiome composition between the control group and the other three experimental groups at the genus level were determined (Figure 5B). The abundance of three genera, including *unclassified_f_Comamonadaceae, Flavobacterium*, and *Legionella*, significantly increased, while that of five genera, including *Aeromonas, Bosea, Devosia, unclassified_f_Rhizobiaceae*, and *Mycobacterium*, significantly decreased in the NIS group compared with those in the CON group. Furthermore, the genus *Thermomonas*, which was detected in the CON group, disappeared in the NIS group. The CEC group significantly increased in the abundance of *Shewanella, Chryseobacterium*, and *Psychrobacter* and decreased in the abundance of *Bosea, Devosia, Mycobacterium, unclassified_f_Rhizobiaceae, Thermomonas*, and *norank_f_Rhizobiales_Incertae_Sedis*, compared with those in the control group. The abundance of the genera *Flavobacterium, unclassified_f_Comamonadaceae, Pseudorhodobacter*, and *Allorhizobium-Neorhizobium-Pararhizobium-Rhizobium* increased, whereas that of the genera *Bosea, unclassified_f_Rhizobiaceae, Mycobacterium*, and *Thermomonas* significantly decreased in the PCH group, compared with those in the control group. A ternary plot was used to visually represent the composition and proportional distribution of the dominant genera in the CON, NIS, and CEC groups (Figure 5C). These results suggest that different AMPs (nisin and cecropin) have different effects on the composition of the gut microbial community in common carp. LEfSe analysis was performed to determine significantly different bacteria among the four groups (Figures 6A,B). We also performed hierarchical clustering heatmap analysis at the species level to reveal the changes in the entire gut microbial community structure of common carp caused by nisin, cecropin, and *P. chinense* (Figure 6C).

### Effects of nisin, cecropin, and *P. chinense* on Immune-Related Gene Expression

**Figure 7:**
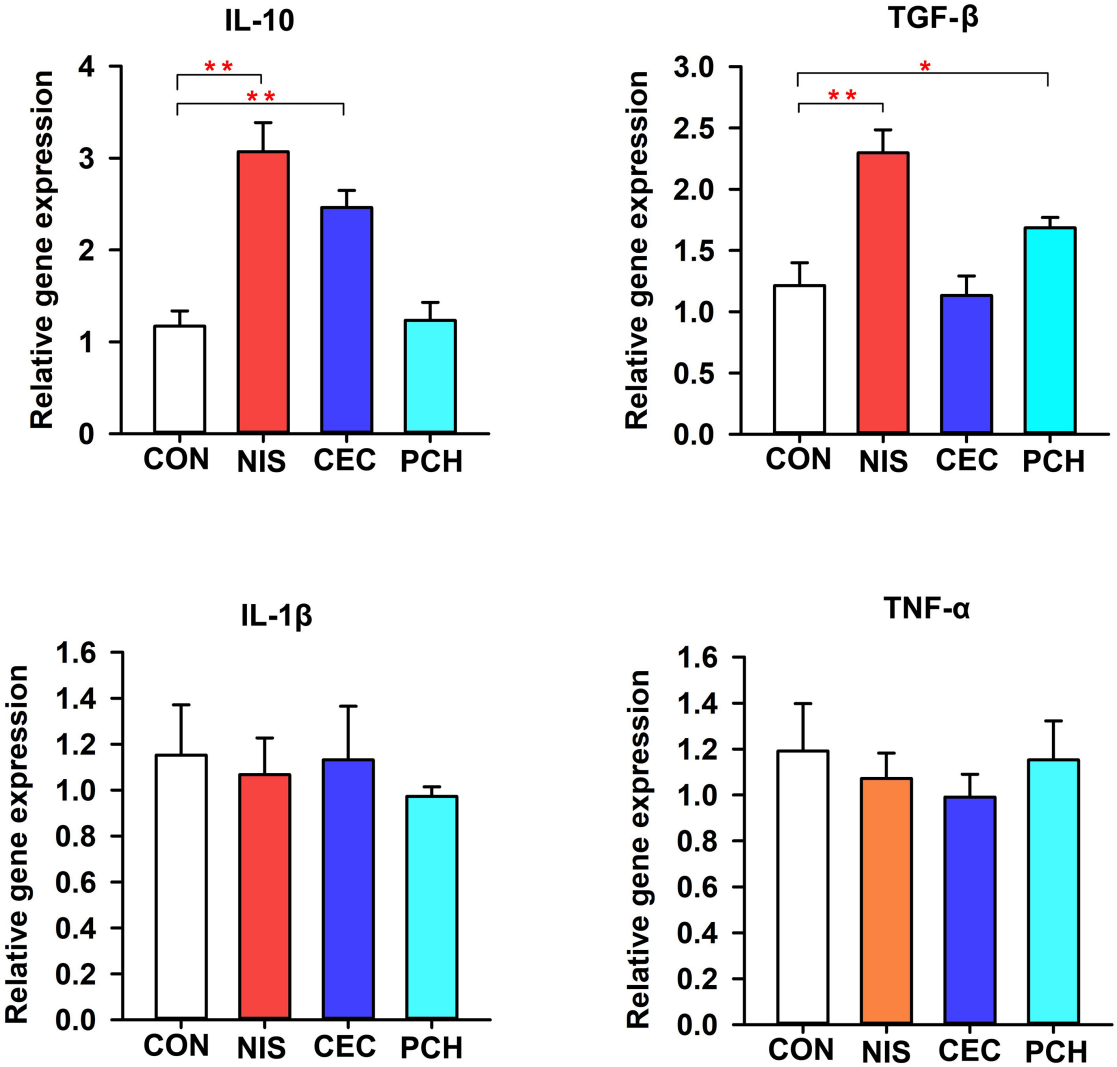
Expression of immune-related genes in the intestine of the common carp in the four groups. The results were normalized against those of β-actin. Data are expressed as mean ± SD (*n* = *6*). A significant difference compared with the control (CON) group is indicated by an asterisk. Statistical analysis was performed using one-way ANOVA with Dunnett's test (*p < 0.05, **p < 0.01).

The expression levels of immune-related genes (TNF-α, IL-1β, IL10, and TGF-β) in the four groups were determined using qRT-PCR. As shown in Figure 7, the expression levels of IL10 significantly increased in the NIS and CEC groups compared with that of the control group. By contrast, the PCH group did not show any significant differences. The NIS and PCH groups showed significantly higher TGF-β expression levels than the CON and CEC groups. There were no significant differences detected between the CON group and the three experimental groups in terms of IL-1β or TNF-α gene expression levels.

### Effects of nisin, cecropin, and *P. chinense* on Gut Digestive Enzyme Activity

Digestive enzyme activities in the intestines of common carp were determined and compared between the control and the three experimental groups (Table 2). Intestinal amylase activities in all groups were not significantly different. The NIS and CEC groups showed significantly higher protease activity than the CON group. The lipase activities in the CEC and PCH groups significantly decreased and increased, respectively, compared with that of the CON group.

**Table 2:** Comparative analysis of digestive enzyme activities of common carp in the four groups.

**Items**	**CON**	**NIS**	**CEC**	**PCH**
Amylase (U/mg)	0.42 ± 0.07	0.36 ± 0.11	0.38 ± 0.11	0.43 ± 0.08
Protease (U/mg)	14.72 ± 1.96	31.09 ± 2.27^a^	22.99 ± 3.93^a^	15.02 ± 1.93
Lipase (U/mg)	25.51 ± 2.17	27.90 ± 4.44	20.66 ± 1.36^a^	32.26 ± 5.09^a^

a *Indicates significant differences compared with the CON group. Statistical analysis was performed using one-way ANOVA with Dunnett's test*. *(n = 6 fish for each group; p < 0.05)*.

## Discussion

Application of immunostimulants, including prebiotics (oligosaccharides, polysaccharides, and nutrients), probiotics, herbs, and AMPs as feed additives is occurring frequently in aquaculture (2). Studies have reported that supplementation with probiotics and prebiotics, herbs, and AMPs, individually or together (synbiotics) in the diet, could have beneficial effects on survival, growth, gut microbiota, immunity, and disease control in farmed fishes (32). However, in some cases, no significant differences in the growth or survival indices of fish were detected at the end of the experimental trials, and overdoses of these substances may have negative effects on the performance of fishes (12, 33). The exact mechanisms of these dietary immunostimulants on fish health status are unknown. It has been speculated that prebiotics can affect immune parameters via the production of short-chain fatty acids (SCFAs) following gut microbial fermentation. SCFAs can regulate innate immune activities by binding to G protein-coupled receptors on immune cells (34). Herbs, which contain various bioactive compounds, have the ability to regulate many biological processes in fish, and some also demonstrate antimicrobial properties (18). Fish intestinal microbial communities are the main factors affecting the fermentability and functionality of the dietary immunostimulants. Therefore, it is critical to investigate the effects of immunostimulants on the intestinal microbial communities of specific fish species for their rational use in aquaculture.

AMPs that can perform immunomodulating functions, endotoxin neutralization, and induction of angiogenesis, are important components of the innate immune system of hosts and are widespread in nature (35). Various studies have investigated the effects of AMPs on growth, performance, antioxidant and immune responses, and disease resistance in poultry and livestock when used as feed additives, and many promising results have been obtained (36). It has been shown that the beneficial effects of AMPs on the growth performance of animals are mainly due to antimicrobial and immunomodulatory activities (37). A study by Ren et al. reported that dietary composite AMPs increased T-cell populations, stimulated the proliferation function of T cells, and decreased the percentage of apoptotic spleen cells in weaning piglets (38). Shan et al. found that lactoferrin could improve the proliferation of spleen lymphocytes in the peripheral blood and effectively increased the concentration of serum antibodies and IL-2 levels in weaning piglets (39). AMPs generated from pig and rabbit systems can improve the intestinal mucosal immunity in broilers (40, 41). Dietary AMPs A3 and P5 enhance the total tract digestibility of weaning piglets and broilers toward crude protein and dry matter, and gross energy production (42). Wang et al. found that dietary AMP sublancin could increase villus height in the duodenum, as well as induce a higher villus-height-to-crypt-depth ratio in the jejunum of broilers (43). AMPs as feed additives can also improve the health of host animals by regulating the intestinal microbial ecology, including suppressing the growth of harmful microorganisms such as *Clostridium* and promoting the growth of beneficial microorganisms such as *Lactobacillus* and *Bifidobacterium* (44).

Compared with results observed in poultry and livestock, the effects of AMPs in aquaculture are understudied and may be more complicated owing to their reduced stability in water and direct interaction with the gut microecology in stomachless fish. Liu et al. reported that feeding grass carp with compound AMP-containing diets improved growth performance, as well as antioxidant and digestive capabilities, enhanced the expression of immune-related genes, and increased resistance to *Aeromonas hydrophila* challenge (12). Li et al. also claimed that AMPs supplemented into the diet can promote growth performance, improve oxidation resistance and immunity, increase digestive enzyme activity, and improve the intestinal morphology of juvenile largemouth bass (*Micropterus salmoides*) (24). AMP has been found to reduce serum triglyceride levels, improve immunity, and enrich the oxidation resistance of common carp (45). Recently, Dai et al. reported that cecropin AD could improve intestinal immunity and enhance the disease resistance of juvenile turbot (*Scophthalmus maximus* L.) (46). Thus, these studies clearly demonstrate that AMPs have great application potential in aquaculture. A large variety of microbes with roles in nutrition, metabolism, and immunity can colonize the fish intestine. However, little is known about the effects of AMPs as feed additives on the gut microbiome of fish. Systematically addressing this issue may be helpful in revealing underlying mechanisms of the beneficial effects of AMPs on fish growth performance.

This study investigated the effects of two agriculturally important AMPs (nisin and cecropin) on the gut microbiome of common carp. Our results suggest that both nisin and cecropin alter the composition of the gut microbiome of common carp; however, the changes caused by these two AMPs are distinct from one another. The PCoA plot and hierarchical clustering tree revealed that the nisin group clustered away from the cecropin and control groups, whereas the cecropin group was located close to the control group in the PCoA plot and was clustered in the same branch of the tree (Figure 3). We speculate that the different effects of these two AMPs on the microbial communities of common carp may be attributed to their different antimicrobial spectra and mechanisms of action.

Chinese herbs have been used as traditional medicines to treat human diseases for thousands of years in East Asia. Various functional components from herbs, including minerals, alkaloids, flavonoids, vitamins, fatty acids, polysaccharides, and proteins, have been shown to have immunostimulatory, antitumor, antiviral, and antibacterial activities (2). The application of herbs with immune-stimulating functions in aquaculture has recently drawn increased attention. Several studies have demonstrated that herbs can enhance phagocytosis in fish. The phagocytic activity of blood leukocytes in crucian carp was enhanced after feeding with Chinese herbs (*I. indigotica, R. officinale, L. japonica*, and *A. paniculata*) (2). A mixture of *Astragalus* and *Lonicera* extracts increased the respiratory burst and phagocytic activities of blood phagocytes and plasma lysozyme activity in Nile tilapia, and increased resistance to *A. hydrophila* infection (47). Both *Angelica sinensis* and *A. membranaceus* showed the ability to activate the immune systems of rainbow trout, catla carp, Mozambique tilapia, common carp, and large yellow croaker (2). Studies on the effects of herbs on the gut microbiota community in fish are relatively limited. In this study, the effects of *P. chinense* on the gut microbiota of common carp were investigated, and it was found that it significantly altered the composition of the microbial community. The *P. chinense* group partially overlapped with the nisin group in the PCoA plot and was located in the same branch of the hierarchical clustering tree (Figure 3). This result indicates that *P. chinense* has a similar effect on the gut microbiota community of common carp to that of nisin.

The predominant bacterial phyla in common carp detected in this study were Proteobacteria, Bacteroidetes, Actinobacteria, Firmicutes, and Fusobacteriota, consistent with the results of previous study (48). After consuming nisin- and *P. chinense-*containing feed, the abundance of *Flavobacterium* was significantly increased in the gut microbiota community of common carp. *Flavobacterium* is widespread in soil and freshwater, and several species of this genus are known to cause diseases in freshwater fishes (49). The relative abundance of *Shewanella* was significantly increased in the cecropin group, compared with that of the control group. *Shewanella* is an opportunistic pathogen in fish and can damage the intestinal immune system of its host (50). The relative abundance of *Aeromonas* was reduced in the three experimental groups compared to that in the control group. Most of the 14 described species in *Aeromonas* genera have been associated with diseases, including bacterial enteritis, septicemia, and furunculosis (51). Based on these results, we conclude that nisin, cecropin, and *P. chinense* can alter the abundance of some pathogens, including opportunistic pathogens, in the gut microbial community of common carp.

In this study, the effects of nisin, cecropin, and *P. chinense* on the expression levels of four cytokine genes (TNF-α, IL-1β, IL10, and TGF-β) were also determined. Cytokines are key mediators of many biological processes, including tissue repair, blood cell production, cell growth, immune response, and inflammation (52). TNFα and IL-1β are typical proinflammatory cytokines, whereas TGFβ and IL10 are two anti-inflammatory cytokines. After consuming the nisin-, cecropin-, and *P. chinense-*containing feeds, the expression levels of IL-10 and TGF-β were significantly increased, which may indicate that these three substances have anti-inflammatory effects in the gut of common carp. Cecropin and *P. chinense* significantly increased the expression levels of IL-10 and TGF-β, respectively, whereas nisin upregulated the expression of both cytokines simultaneously. Similar effects of dietary AMPs and herbs on fish health have been reported by other research groups. For example, dietary turmeric has antistress, antioxidant, and anti-inflammatory effects in common carp (26). Dietary *Ginkgo biloba* extracts can upregulate the expression of antioxidant genes, immune-related genes, and anti-inflammatory cytokines IL-10 and TGF-β in hybrid grouper (53). Eucalyptol has also shown anti-inflammatory effects on common carp exposed to ambient copper (54). Expression levels of IL-10 and TGF-β are significantly upregulated in grass carp challenged with *A. hydrophila* after feeding diets supplemented with compound AMPs (12). However, in some cases, contradictory results are reported. For example, when supplemented as feed additive, *Rehmannia glutinosa* upregulates the expression of IL-1β, TNF-α, and iNOS, and downregulates the expression of IL-10 and TGF-β (25). Similarly, when guava leaves were added to the diet of *Labeo rohita*, expression levels of IL-1β and TNF-α were significantly increased, whereas levels of IL-10 and TGF-β were significantly decreased (55). These contradictory effects of immunostimulants on the expression levels of cytokines in fishes may have many causes. It has been shown that both AMPs and herbs are effectors of innate and adaptive immunity, capable of modulating pro- and anti-inflammatory responses, and that they also have chemotactic activity (9, 18). AMPs and herbs with different bioactive compounds may have different targets and mechanisms to regulate inflammatory responses. AMPs and herbs may also indirectly affect the inflammatory responses in the gut of fishes by altering the gut microbiota composition, which is known to affect gut mucosal immunity. Our study supports the assertion that different AMPs and herbs have different effects on the gut microbial community of fishes. Additionally, the supplied concentration and experimental conditions may also affect the function of immunostimulants in inflammatory responses in fishes.

Although dietary supplementation of nisin, cecropin, and *P. chinense* did not produce any significant improvement in growth performance in this study, all three demonstrated the ability to alter specific digestive enzyme activities, which may mean that supplementation of these substances in feed can affect the digestive ability of common carp. Other studies with similar results have been reported. When supplemented at a concentration of 3 g/kg in the diet, aqueous extract of spade flower (*Hybanthus enneaspermus*) significantly improves the activities of amylase, lipase, and protease in rohu (56). Some digestive enzyme activities in the stomach and intestine of Japanese seabass were found to be significantly increased when dietary supplementation of a Chinese herbal medicine mixture was added (57). AMPs as feed additives may increase protease and lipase activity, as well as reduce the amylase activity in largemouth bass at specific concentrations (24). Notably, most of these studies describe only the effects of immunostimulants on the digestive enzyme activities, without underlying mechanism analysis. We speculate that immunostimulants as feed additives may affect digestive enzyme activity directly by regulating the corresponding gene expression or indirectly by altering the gut microbiota composition, which plays important roles in host nutrition. However, understanding the molecular mechanisms underlying the effects of supplemented immunostimulants on digestive enzyme activities requires future experiments to be conducted.

In summary, our current results suggest that nisin, cecropin, and *P. chinense*, when supplemented as feed additives, affect the diversity, abundance, and even the structure of the gut microbial community in common carp. Additionally, these three additives also show anti-inflammatory effects, and enhance specific digestive enzyme activity in the gut of common carp. For large-scale use of AMPs and Chinese herbs in aquaculture, further investigations into *in vitro* and *in vivo* toxicological tests, digestibility by fishes, and stability in the aquatic environment should be conducted. Additionally, considering that a given AMP or herb commonly shows various effects on different fish species, identifying the most effective substances for use in a specific fish species is also important.

## Data Availability Statement

The datasets presented in this study can be found in online repositories. The names of the repository/repositories and accession number(s) can be found in the article/Supplementary Material.

## Ethics Statement

The animal study was reviewed and approved by Southwest Medical University Ethics Committee.

## Author Contributions

Experiments were conceived and designed by XG and performed by FK, PX, YY, LY, AG, JY, and JZ. Data were analyzed by XG. The initial draft of the manuscript was written by XG and critically revised by LL and QW. All authors have read and approved the final manuscript.

## Funding

This work was supported by the China Postdoctoral Science Foundation (2020M683364), the Department of Science and Technology of Sichuan Province (2020YJ0129), the Collaborative Fund of Science and Technology Agency of Luzhou Government and Southwest Medical University (20YKDYYJC0030), and the Science Fund Project of Southwest Medical University (2019ZQN024 and 2020ZRQNA007).

## Conflict of Interest

The authors declare that the research was conducted in the absence of any commercial or financial relationships that could be construed as a potential conflict of interest.

## Publisher's Note

All claims expressed in this article are solely those of the authors and do not necessarily represent those of their affiliated organizations, or those of the publisher, the editors and the reviewers. Any product that may be evaluated in this article, or claim that may be made by its manufacturer, is not guaranteed or endorsed by the publisher.
