# Supplementary material for: Effects of Nisin, Cecropin, and Penthorum chinense Pursh on the Intestinal Microbiome of Common Carp (Cyprinus carpio)
Source: Front Nutr. 2021 Oct 21;8:729437. doi: 10.3389/fnut.2021.729437 (PMC8566669; doi:10.3389/fnut.2021.729437)
Supplement: Supplementary file 1 [file Data_Sheet_1.docx]

**Table S1. Information of valid reads of 24 samples in this study.**

| Sample | Sequence number | Base number | Mean length | Minimal length | Maximal length | Coverage (%) |
| --- | --- | --- | --- | --- | --- | --- |
| CON1 | 53489 | 22141826 | 414 | 401 | 430 | 99.96 |
| CON2 | 57029 | 24383340 | 426 | 402 | 430 | 99.94 |
| CON3 | 74812 | 31240389 | 418 | 382 | 431 | 99.90 |
| CON4 | 69034 | 28118267 | 407 | 297 | 431 | 99.89 |
| CON5 | 54878 | 23361059 | 426 | 402 | 430 | 99.92 |
| CON6 | 65389 | 27577477 | 422 | 342 | 436 | 99.89 |
| NIS1 | 52592 | 22219583 | 422 | 370 | 444 | 99.95 |
| NIS2 | 49274 | 21006956 | 426 | 402 | 430 | 99.92 |
| NIS3 | 57069 | 24234922 | 425 | 262 | 444 | 99.93 |
| NIS4 | 67003 | 27995069 | 418 | 381 | 430 | 99.89 |
| NIS5 | 51539 | 21870729 | 424 | 401 | 430 | 99.91 |
| NIS6 | 38689 | 16451041 | 425 | 342 | 430 | 99.96 |
| CEC1 | 59835 | 25358092 | 424 | 392 | 478 | 99.89 |
| CEC2 | 64827 | 27749466 | 428 | 402 | 431 | 99.92 |
| CEC3 | 46519 | 19891937 | 428 | 400 | 438 | 99.88 |
| CEC4 | 39839 | 17059604 | 428 | 403 | 430 | 99.88 |
| CEC5 | 44819 | 19206844 | 428 | 402 | 432 | 99.88 |
| CEC6 | 64307 | 27250343 | 424 | 348 | 431 | 99.91 |
| PCH1 | 45078 | 19157167 | 425 | 402 | 430 | 99.89 |
| PCH2 | 42476 | 18044774 | 425 | 403 | 431 | 99.93 |
| PCH3 | 50348 | 21316370 | 423 | 401 | 498 | 99.88 |
| PCH4 | 31527 | 13353362 | 424 | 402 | 431 | 99.94 |
| PCH5 | 36489 | 15351049 | 421 | 402 | 430 | 99.91 |
| PCH6 | 65532 | 26898037 | 410 | 342 | 432 | 99.90 |


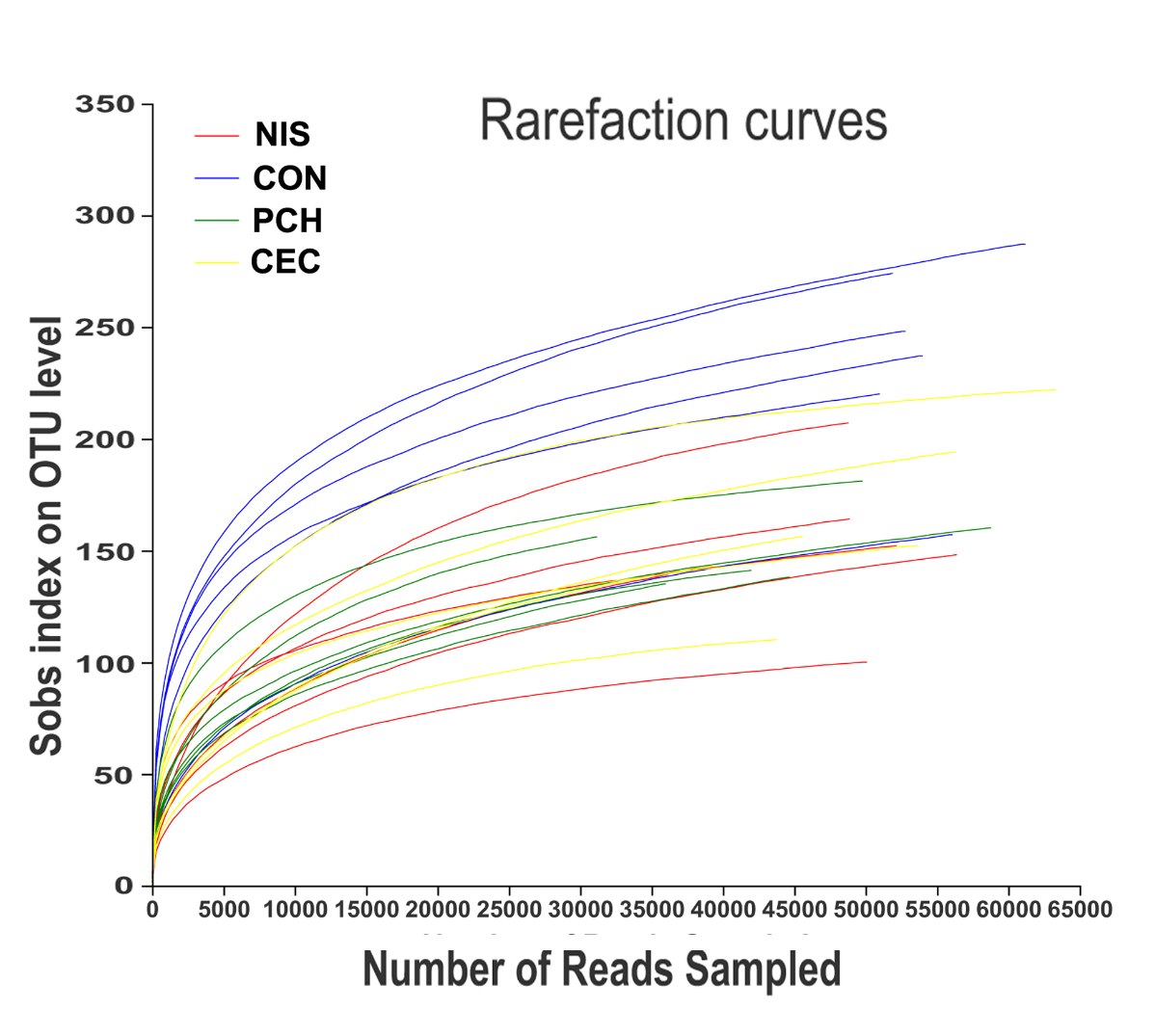


**Figure S1.** **Rarefaction analysis of different samples.** Rarefaction curves of the different samples performed high-throughput sequencing. OTUs are identified using 97 % cutoffs. Total sample richness is presented as the richness estimator Sobs index. The curve was generated by plotting the number of valid sequences for each sampled fish against the Sobs index of OTUs.


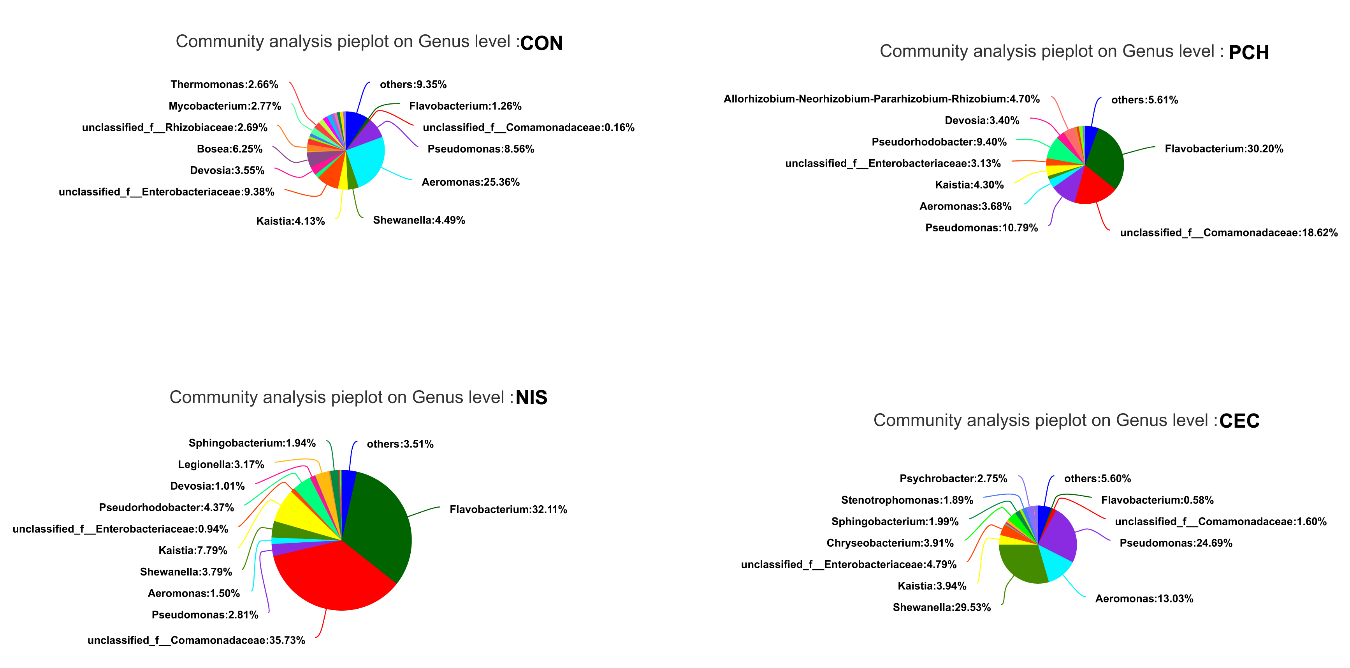


**Figure S2. Distribution of bacterial genus among the four groups.** The pie diagrams show the bacterial composition of the control group, the *P. chinense* group, the nisin group, and the cecropin group, and the relative abundances of the bacterial genus in each group are shown. Others represent bacteria with less than 0.1% abundance at the genus level.


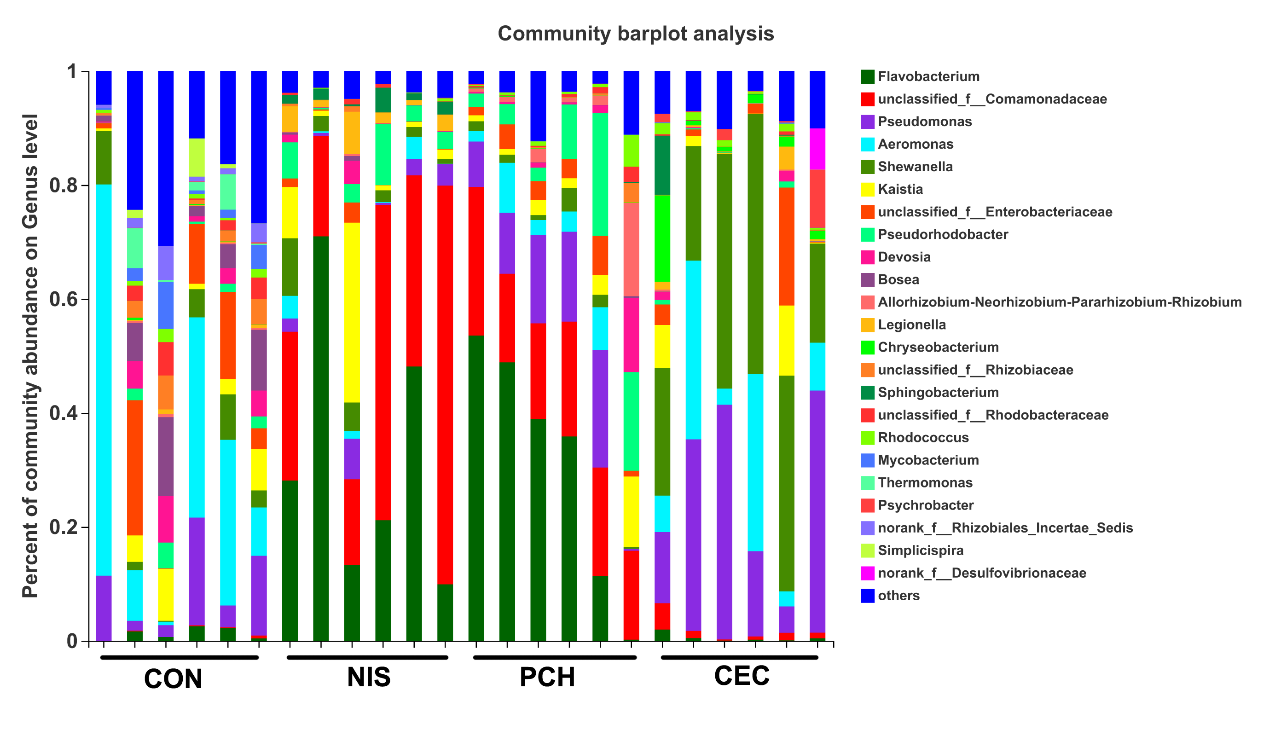


**Figure S3. Relative abundance of bacteria at genus levels in the four groups**
